# Visual questionnaire survey to apply design possibilities in relation to planting enclosure in five vest-pocket parks

**DOI:** 10.1016/j.mex.2022.101638

**Published:** 2022-02-26

**Authors:** Maryam Naghibi, Mohsen Faizi, Ahmad Ekhlassi

**Affiliations:** School of Architecture and Environmental Design, Iran University of Science and Technology, Tehran 1684613114, Iran

**Keywords:** Visual questionnaire, Preference, Leftover space, Pocket park

## Abstract

The method presented in the article is helpful for analyzing the aesthetical landscape preferences. Using visual and physical plant combinations in small abandoned spaces, this study seeks to discover what citizens prefer in terms of coherence, legibility, and refuge? The procedure relies on visual questioning to get residents’ opinion based on three criteria: legibility, coherence, and refuge. Specifically, it aims at exploring the relation of three landscape variables with the enclosure. Participants gave the lowest ratings for refuge in visually open & physically open environments, according to the data. In visually open environments, the participants' perception of coherence does not vary significantly. When compared to other characteristics (coherence and legibility), the research of spatial configurations of vest-pocket parks shows that enclosure is more important in terms of refuge. Finally, the significant difference between object-focused parks and others indicated the critical design process of this type of pocket park.

• Visual questionnaire survey is helpful for analyzing the aesthetical landscape preferences.

• A photomontage method could apply design possibilities in relation to planting.

## Graphical abstract

**Figure:**
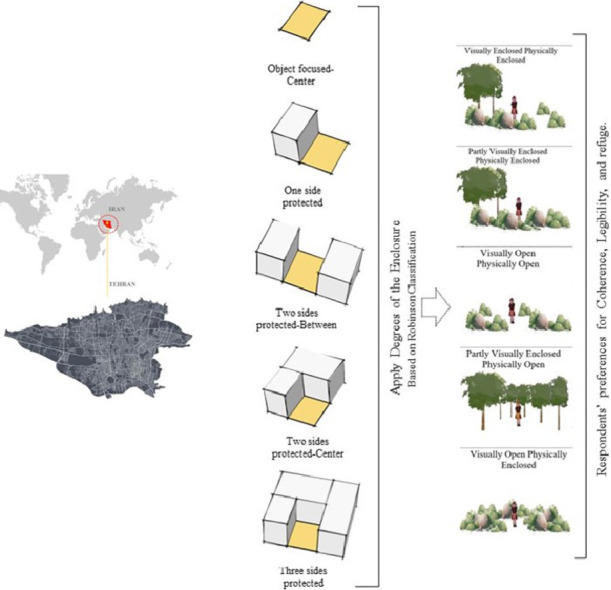

Specifications table

**Table:** 

Subject Area:	Environmental Science
More specific subject area:	Landscape
Method name:	Visual questionnaire survey
Name and reference of original method:	*NA*
Resource availability:	*NA*

## Background

Books on questionnaire design also do not reveal anything specific regarding visual questionnaires [1], [2], [3]. The sole mention of visuals is by Oppenheim, who uses word to describe projective techniques (indirect techniques). Where direct attitude assessment methods are ineffective, the projective methodology uses visuals to convey the self-image. When it comes to the perception of human relationships, the research is especially useful. Because there were no pre-existing standards or guidelines for constructing the visual questionnaire, it was created by trial and error under certain constraints.

The visual questionnaire was created with the goal of creating a useful research tool for the landscape field. It can also help participants and researchers communicate better by clarifying the nature of the inquiries [4]. The utilization of photographic material allowed for the differentiation of preferences for various natural places [5]. The questionnaire for this study was based on earlier research [6], [7], [8], [9]. By adding, removing, or combining the features of an original photograph, a photomontage approach was employed to produce various images [10]. By modifying the original photographs, twenty-five images with various combinations of the visual and physical enclosure were made to analyze the visual preferences for the different enclosures [11], [12], [13], [14]. Plant species were then employed to model five different visual and physical enclosure combinations.

## Methods

In a mixed approach, this study used descriptive strategies. Also, a questionnaire survey was used to examine esthetic preferences for enclosure, coherence, refuge, and legibility regarding data gathering. This process is divided into five phases. First, the authors surveyed the pocket parks classification, and preliminary interviews with locals led to the case studies. In the second phase, a pilot study was conducted to apply the permeability of enclosure in the context of vest-pocket parks. The first two phases contribute to preparing computer-simulated sceneries that the participants will evaluate for coherence, legibility, and refuge.

**Fig. 1:**
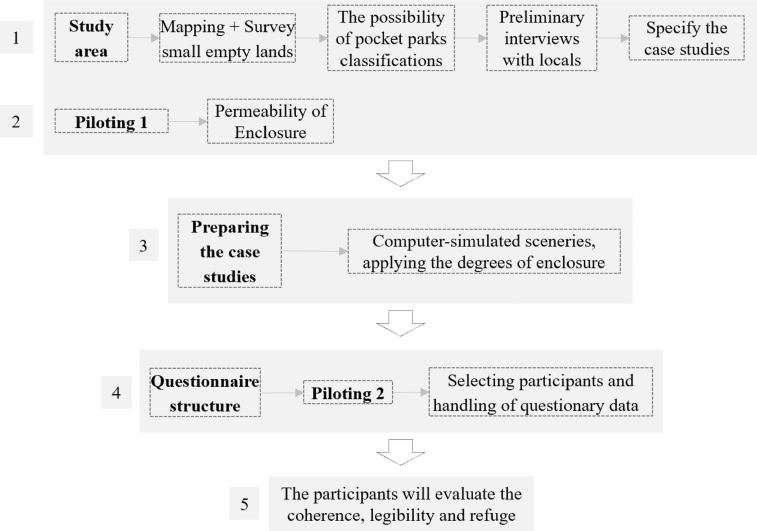
Research procedure.

After the scenes and cases were determined, the final questionnaire was designed. This visual questionnaire was separated into three sections [8] based on the five basic types of permeability [15]. To make sure the sample is representative of the majority population, the first section asked about socioeconomic information and the kind of activity in small parks. The second section asked respondents five questions about their preferences for the five types of spaces’ activities. In terms of the enclosure, the third section consisted of six questions for each pocket park (five typologies). Pocket parks were chosen to represent various degrees of enclosure in this section (Fig. 6). A second pilot study was conducted to ensure that the questions were straightforward. This process leads to the selection of participants and the handling of questionary data. The procedures are illustrated in Fig. 1.

Green places are known to be affected by a participant's attributes [16]. As a result, incorporating a large number of people can help reveal quantitative patterns in a given situation [17]. However, according to Stamps (1996), 40 respondents and 20 photographs are necessary to achieve a suitable effect size of 0.01 at 0.05; so, his study has a sample size of 318 respondents and 25 images. Applications such as WhatsApp, Telegram, and Instagram, and web-sites as: LinkedIn, and email were used to recruit participants.

The data were analyzed using IBM SPSS 26. The statistical tests were used as follows: Chi-square tests were used to identify significant relations between preferences and socio-demographic backgrounds. The Spearman correlation coefficient investigated among coherence, legibility, and refuge. Kruskal-Wallis test was used to find the difference between the Five parks, Mann-Whitney test was used to compare the two pairs of studied parks. Also, Mean Rank and Median in five types of parks were reported.

### Research questions

The current research focuses on small abandoned spaces, focusing on residents' preferences, how planting should be organized to maximize the potential of pocket parks, and the types of activities that might be done in such small parks. By studying user perceptions and evaluating how enclosure on pocket parks may affect inhabitants' preferences, our study contributes to the discussion on diverse visual preferences in leftover places. We used the following crucial questions to achieve this goal: Based on visual and physical plant combinations, what are the preferences of citizens? In terms of coherence, legibility, and refuge, is there any significant difference in vest-pocket parks typology preferences? Following the research questions, the definitions were derived. Coherence: How closely are the components related to one another? and How do simple elements work together to create a more intelligible scene? [18]. When you first walk in, how clear is the scene? Based on the planting, which scene takes the least amount of time to figure out? [7]. Refuge: Which scenario provides the most option for hiding from other people's gazes? [18]. Which scene provides the most comfort? [19]

## Testing area and pilot results

**Fig. 2:**
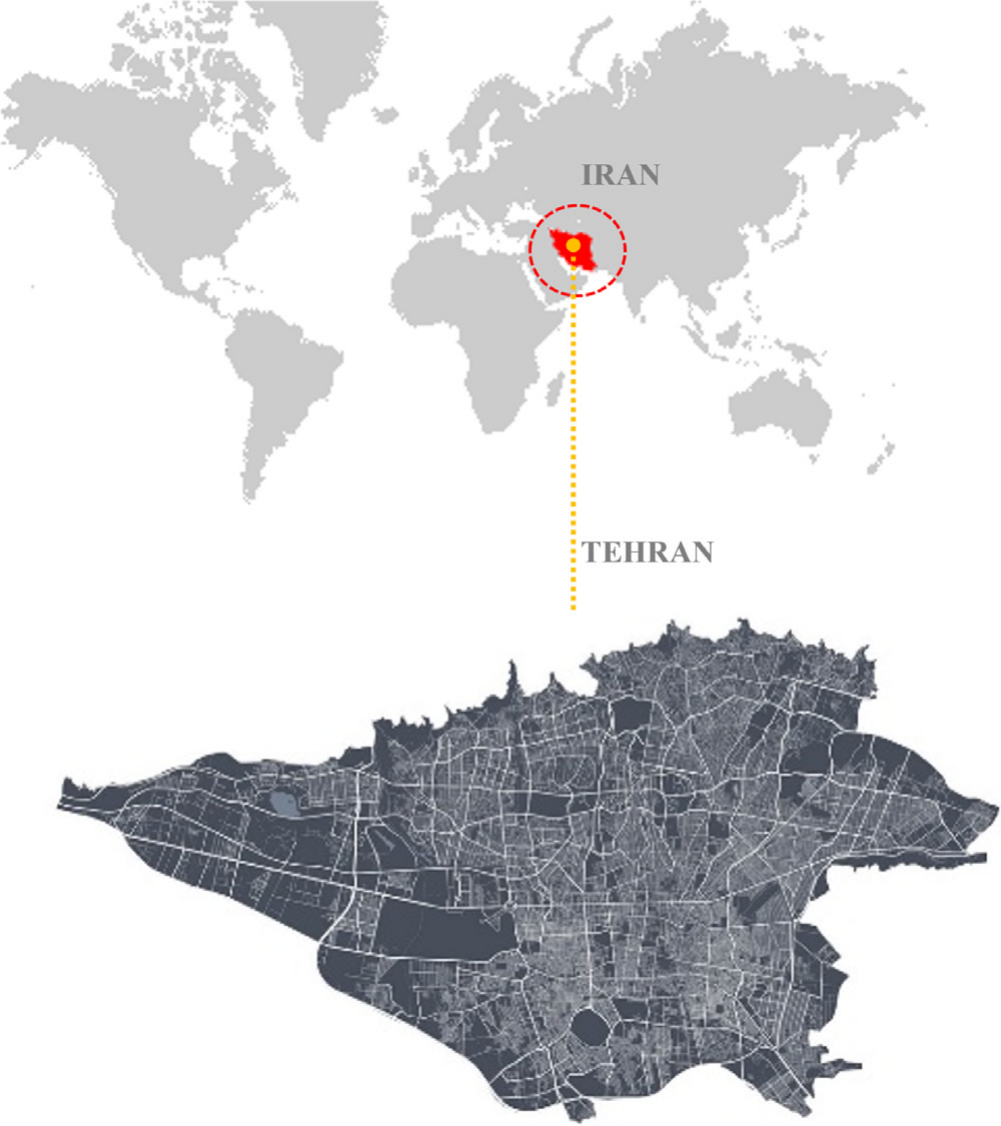
Case study's location.

The research was carried out in Tehran, Iran's capital. Tehran is a megacity in Iran's roughly central north, with major traffic, air pollution, and population issues. The city is situated between the high Alborz Mountains to the north and the middle desert to the south. The researchers chose Tehran as a study region because of the varaity of vacant lands, which fits the study's goal (Fig. 2).

### Sampling design

**Fig. 3:**
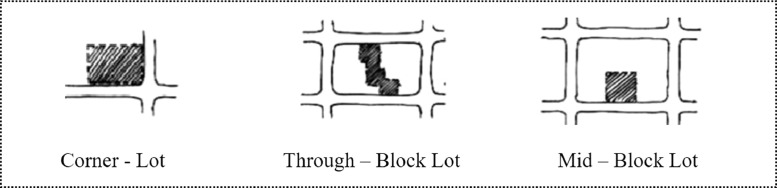
Tree Typical minipark locations [20].

**Fig. 4:**
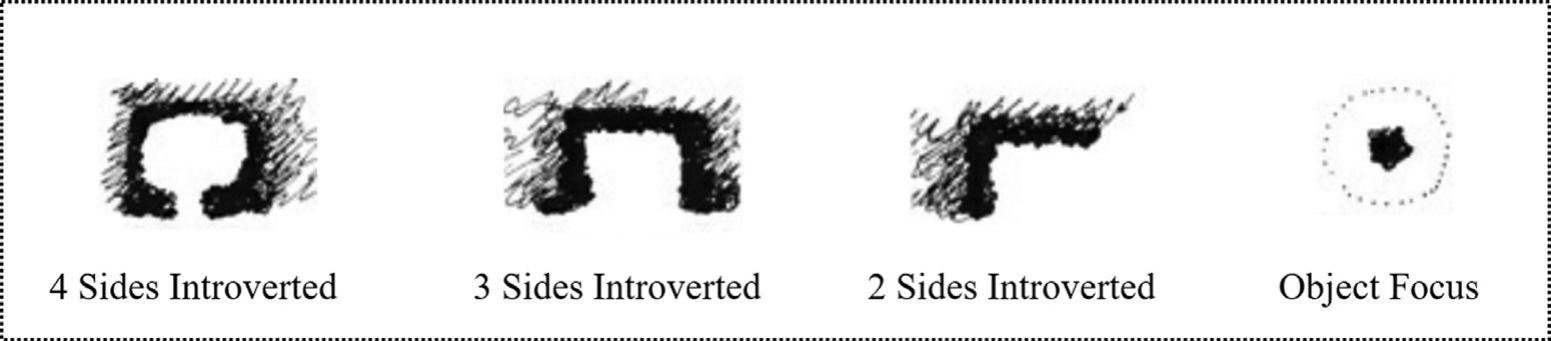
Degrees of the enclosure [15].

The researchers reviewed two areas in Tehran that are considered to have evolved similarly in the first part of the study, with the goal of identifying a list of possible remaining places for investigation. A total of 53 cases of small empty lands were studied. Because of their small size, the majority of them could be photographed in a single shot. In order to categories the pocket parks, this study investigated the minipark location [20], (Fig. 3), degrees of the enclosure [15], (Fig. 4), and the survey conducting with authors.

**Fig. 5:**
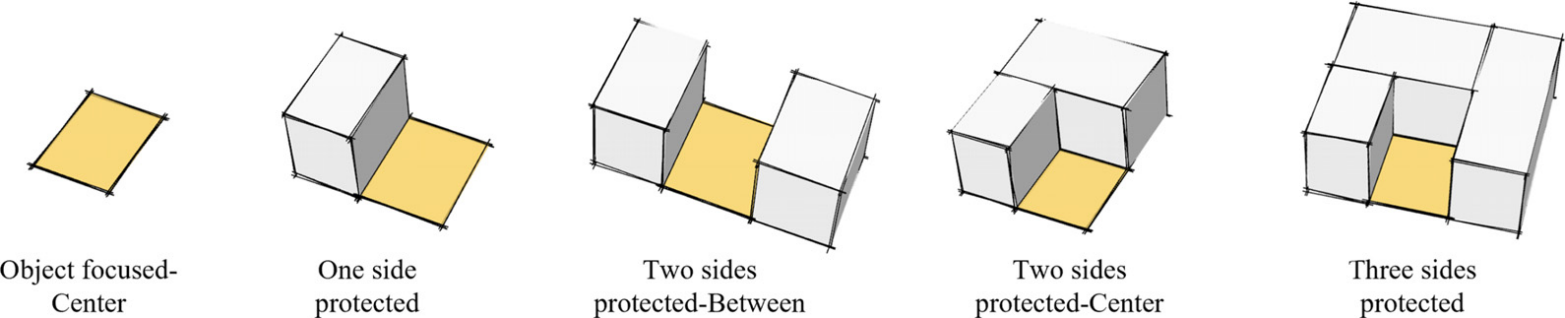
The possibility of pocket parks classifications [21].

Thus, the five categories were established to accommodate abandoned sites that may be converted into pocket parks. The five types of places were then displayed before the original photos were modified (Fig. 5).

In late April 2020, photographs of vacant lands were taken in Tehran. The photographs were shot on the same day using a Samsung-Note 10 phone, with no extra filters or effects [22,23] (approximate height of 160 cm, i.e., from the average adult's view). Lastly, five sample images (a total of 310 photos) were chosen for the survey from among all the photos taken. Spaces having an extent of less than 1 acre [24], adjacent to communities, thought to be vacant lands by neighbors, and remained underutilized were among the criteria used to identify the remaining cases. Preliminary interviews with locals were conducted to include the five locations as leftover spaces in the research. The amount of vegetation in the areas differed from grass, shrubs, and plants to hard surfaces with little vegetation, as presented in Fig. 8, basic scenes.

### Enclosure in the context of vest-pocket parks

In metropolitan environments, enclosure has a significant impact on people's brain reactions and perceptions of their surroundings. Vegetation is one of the most significant ways to create enclosure and shape distinct functional zones in urban parks. Different visual impacts and esthetic sensations are induced by open or confined environments.

Robinson (2004) has demonstrated that there are various types of enclosures from a visual and physical standpoint when it comes to the diversity and multifunctionality of plants [25]. This is known as the Permeability of Enclosure, and there are five different types: visually & physically enclosed, partly visually enclosed & visually enclosed, partly visually enclosed but physically open, visually open but physically enclosed, visually open but physically enclosed, and both visually & physically open.

### Piloting 1: determine the permeability of enclosure

The piloting was launched in April 2020. A total of 20 professionals and non-professionals analyzed the questionnaire for the Permeability of Enclosure, which has five main types. All five primary forms of Robinson's categorization were differentiated in testing interviews.

The purpose of this study is to look into the correlations between three preference criteria and park typology. Legibility, coherence, and refuge were among the several preference variables. It contributes to the enclosure being prioritized as a predictive variable for landscape choice when developing future interventions. Five distinct scenarios for each location were created based on Robinson's visual and physical enclosure combinations. As a result, the design criteria for these places were derived from diverse combinations of visual and physical plants, as well as varying enclosure and openness levels.

### Preparing the case studies

Each site's additional features were illuminated with Adobe Photoshop, and the relevant photos were matched to the background details. The case study was then added to the visual questionnaire. As illustrated in Fig. 7, to apply degrees of the enclosure, each type follows the same process. The background scene presents participants with a sense of direction. The backgrounds were kept since the buildings, and other elements were likely to influence the space's readability. After removing additional elements, background scenes' opacity decreased to reduce confusing parts in the experiment.

An online questionnaire presented with twenty-five scenarios (Fig. 8). Residents were asked to express their visual preferences to investigate the relationships between user needs, design criteria, and user evaluation. User preference was used to test the three variables of coherence, legibility, and refuge as independent variables. First, participants were questioned for basic socio-demographic characteristics, park usage, and possible uses of these pocket parks.

For questions about coherence, refuge, and legibility, the participants were asked to choose the favorite scene.

### Piloting 2: determine the accuracy of completion

Based on Bateman et al. research, additional pilot testing is required to refine the attributes [26]. Thus, before implementing the final questionnaire, this study conducts a comprehensive survey and ensure if the participants comprehend the questions correctly. The pilot process started in May 2020. The results of 20 questionnaires were examined for return rate and accuracy of completion.

Therefore, the final questionnaire survey took place from May 22nd to June 12th, 2020. As a result, a 25-image online questionnaire was used to examine esthetic preferences for enclosure, coherence, refuge, and legibility.

## Data analysis

IBM SPSS 26 was used to conduct the statistical analysis of the data (Table 1). Cronbach's alpha test was used to determine the lower bound for the survey's true reliability. The constructs' reliability ranges from 0.86 to 0.87, indicating an adequate level of reliability (Table 2).

Various analysis techniques, such as descriptive statistics (Frequencies and Descriptive) and correlation tests, were used to analyze the data. Chi-square testing was used in SPSS to investigate the relationship between residents' preferences for enclosure in pocket parks and their sociocultural backgrounds (Table 3).

Chi-square (C) testing was used to find significant associations between participants' preferences and their socio-demographic backgrounds. The impacts of respondents' socio-cultural demographics (age, gender, occupancy, professions, and education) on planting enclosure preferences were statistically significant, as shown in Table 4. Professionalism has the strongest links to coherence, legibility, and refuge, according to the findings.

The descriptive analysis will be done based on each association between the aesthetical perception of participants and their socio-cultural backgrounds. Concerning the issue of people's attitude towards landscape from the visual representation of the material, such as pictures, diagrams, and flow charts, the method, and procedures presented in this article are useful for several research fields, as discussed in our initial study.

The results of the Spearman correlation coefficient showed a positive and significant relationship between Coherence, Legibility, and the refuge, in all five parks (*p* > 0.001). This finding indicated that there is no difference in terms of the relationship between coherence, legibility, and refuge between different parks (Table 5). According to the reported coefficients, the highest correlation coefficient is between coherence and legibility in type 03 park (*r* = 0.586), the highest correlation coefficient is between coherence and refuge in type 04 park (*r* = 0.511), and the highest correlation coefficient is between legibility and refuge in park type 05 (*r* = 0.511) (Table 5).

Mean rank and median mean coherence, legibility and refuge are reported in each studied park (Table 6). The results of the non-parametric Kruskal-Wallis test showed that there was a significant difference between the Five parks in terms of Coherence and Legibility (*p* > 0.001), but there was no significant difference in terms of refuge (*p* = 0.173).

**Fig. 6:**
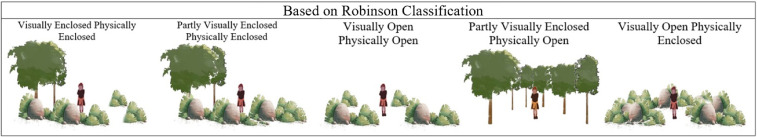
Permeability of enclosure [15].

**Fig. 7:**
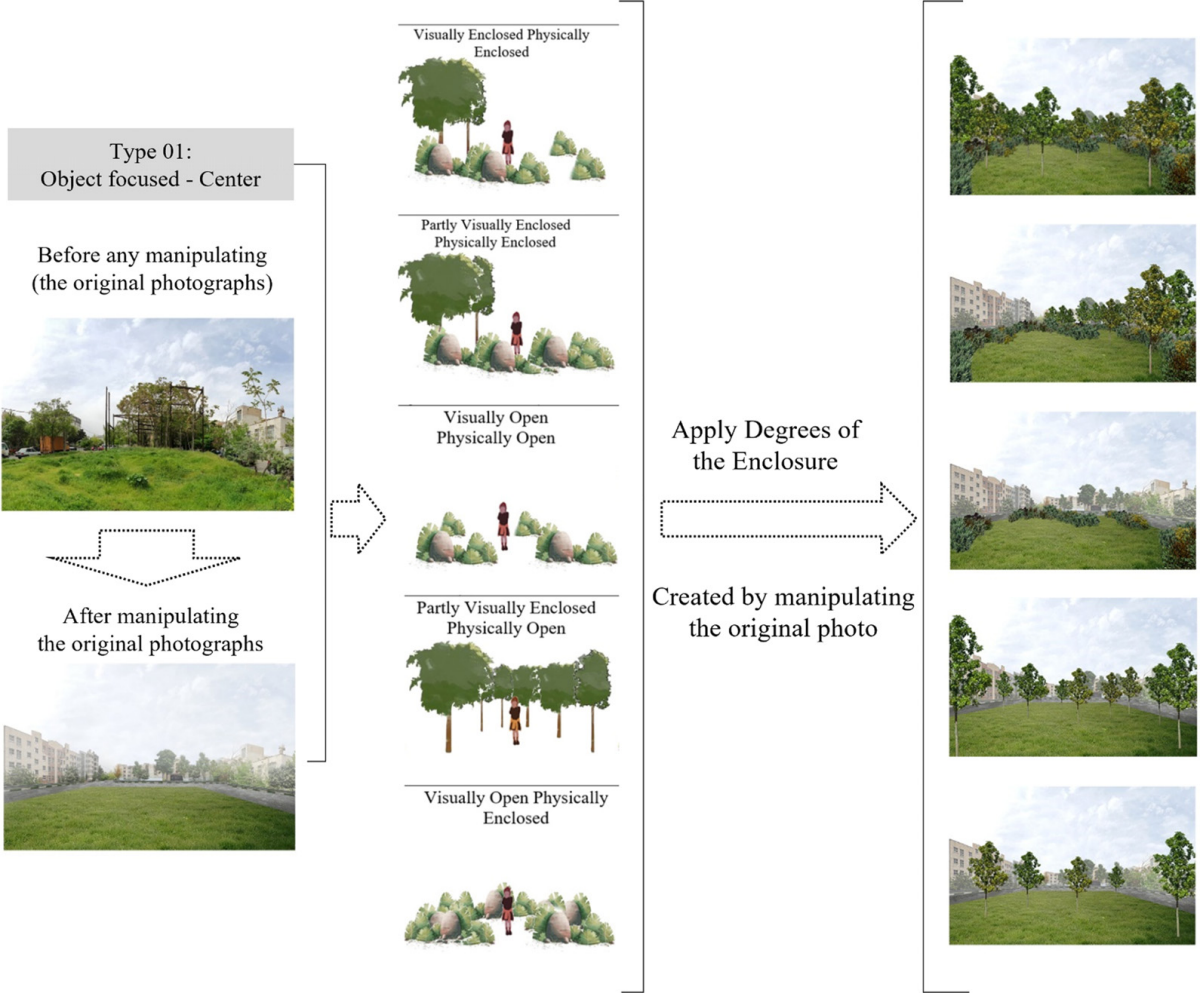
The illustration of computer-simulated sceneries, applying the degrees of enclosure [21].

**Fig. 8:**
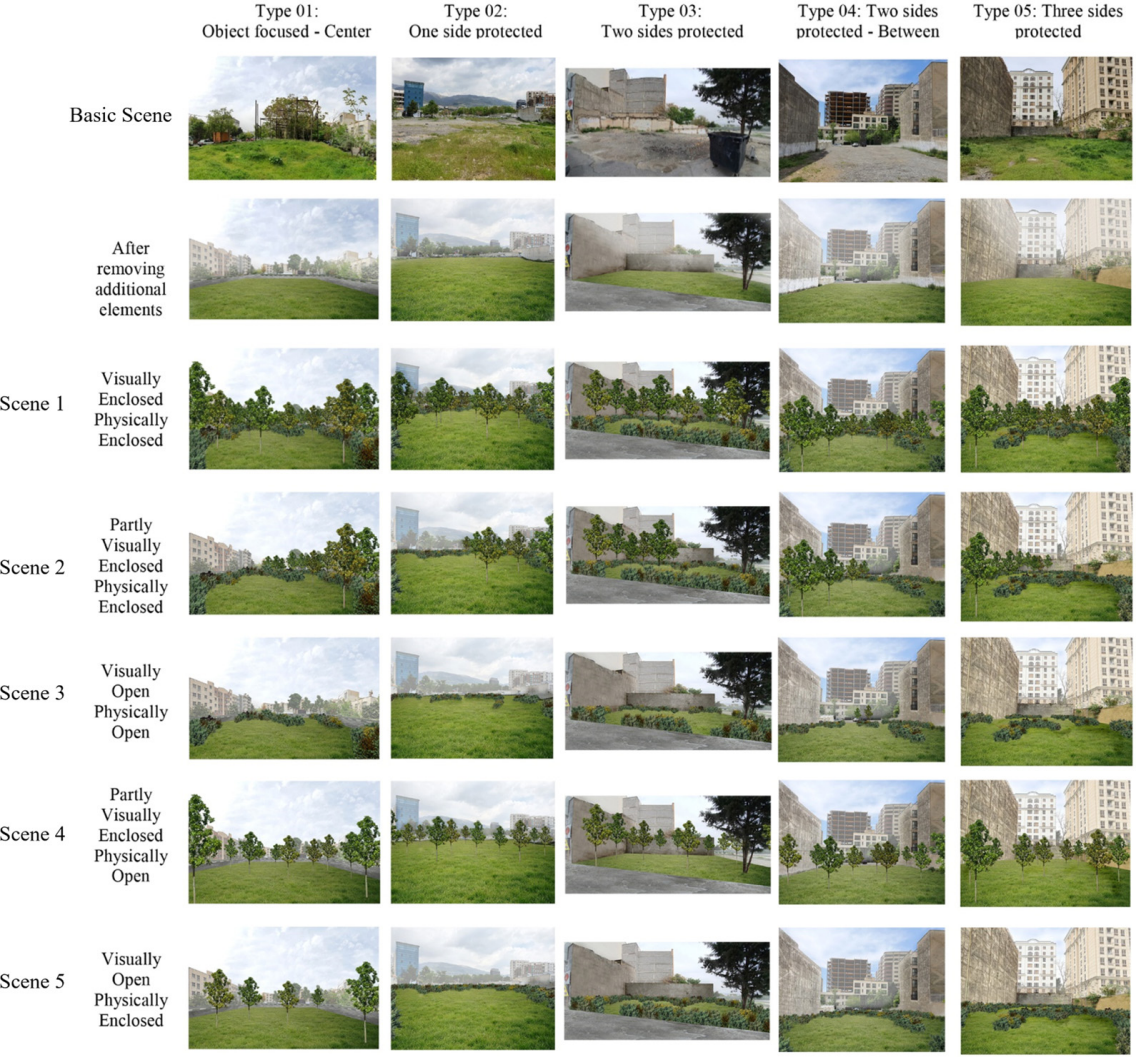
The 25 computer-generated sceneries represent various levels of enclosure made by planting.

**Fig. 9:**
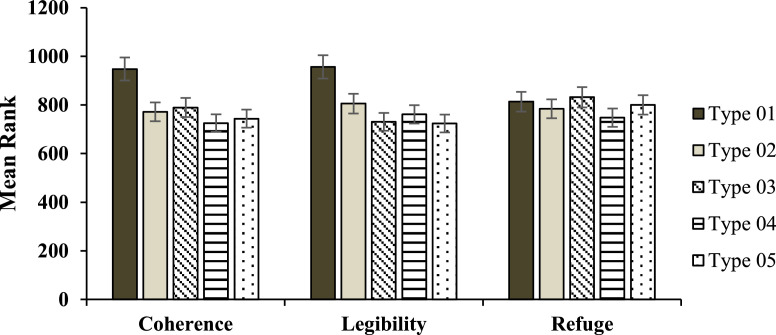
Mean Rank in five types of parks in coherence, legibility and refuge.

The non-parametric Mann-Whitney test was used to compare the two pairs of studied parks in terms of legibility, coherence, and refuge: Coherence was significantly higher in Type 01 than the others (Type 02–05) (*p* > 0.001). Also, in terms of Coherence, there is no significant difference between Type 02, Type 03, Type 04, and Type 05 (*p* > 0.05). Type 01 was significantly higher legibility than the other four parks (Type 02–05) (*p* > 0.001). Also, the legibility of Type 02 was significantly higher than Type 03 and Type 05 (*p* > 0.05). No significant difference was found between Type 02 and Type 04, also between Type 03, Type 04, and Type 05 in terms of Legibility (*p* > 0.05). In terms of refuge, Type 03 park was significantly indicated than Type 04 (*p* > 0.05). In other two-way comparisons, no significant difference was found between parks (*p* > 0.05) in terms of refuge (Table 7 and Fig. 9).

It seems that the design of a park where the pocket park is object-focused will be substantial. Therefore, the elements used in these types should be selected more carefully. Also, the characteristics of plants, crown, height, and density should be considered more than other types.

**Table 1:** Case processing summary.

		N	%
Cases	Valid	309	100.0
	Excluded	0	.0
	Total	309	100.0

a. Listwise deletion based on all variables in the procedure.

**Table 2:** Reliability statistics.

Cronbach's Alpha	Cronbach's Alpha Based on Standardized Items	N of Items
.868	.856	38

**Table 3:** Example of Chi-square Test.

	Value	df	Asymptotic Significance (2-sided)
Pearson Chi-square	48.145a	24	.002
Likelihood Ratio	41.980	24	.013
Linear-by-Linear Association	.006	1	.936
N of Valid Cases	311		

a. 25 cells (71.4%) have expected count less than 5. The minimum expected count is 0.21.

**Table 4:** The Chi-square (C) test analyzed the correlation between participants' esthetic opinions and their socio-demographic backgrounds.

	Type 01	Type 02	Type 03	Type 04	Type 05
**Coherence**					
Age	**	–	****	–	****
Gender	****	****	**	–	–
Education	***	***	***	–	–
Occupation	*	****	*	*	–
Professional	****	**	***	**	*
**Legibility**					
Age	****	***	****	NS	***
Gender	–	–	–	NS	–
Education	*	**	**	NS	*
Occupation	–	**	**	NS	–
Professional	****	*	**	NS	****
**Refuge**					
Age	–	****	****	****	*
Gender	–	–	–	–	–
Education	**	**	*	**	–
Occupation	–	–	–	**	*
Professional	***	****	**	*	****

Significance levels (p-value) show associations between groups: 0.01 ≤ *p ≤ 0.1; **p < 0.05; ***p ≤ 0.01; ****p ≤ 0.001; -, not significant.

**Table 5:** Spearman correlation coefficients among coherence, legibility and refuge.

		Coherence	Legibility	Refuge
Type 01	Coherence	1	0.468***	0.369***
	Legibility		1	0.213***
	Refuge			1
Type 02	Coherence	1	0.435***	0.445***
	Legibility		1	0.294***
	Refuge			1
Type 03	Coherence	1	0.586***	0.446***
	Legibility		1	0.316***
	Refuge			1
Type 04	Coherence	1	0.511***	0.511***
	Legibility		1	0.314***
	Refuge			1
Type 05	Coherence	1	0.559***	0.491***
	Legibility		1	0.389***
	Refuge			1

Note. Significance levels (*p*-value): ***p<0.001.

**Table 6:** Distribution of mean rank and median in five types of parks in coherence, legibility and refuge.

Types of Parks	N	Coherence		Legibility		Refuge	
		Mean Rank	Median	Mean Rank	Median	Mean Rank	Median
Type 01	318	947.98	6.00	956.82	7.00	813.58	4.00
Type 02	318	771.52	4.00	805.63	6.00	784.08	4.00
Type 03	318	789.14	4.00	730.08	6.00	831.66	4.00
Type 04	318	725.43	4.00	761.32	6.00	747.83	4.00
Type 05	318	743.44	4.00	723.65	6.00	800.34	4.00

**Table 7:** Mann-Whitney tests to pairwise comparisons of five types of parks in coherence, legibility and refuge.

pairwise comparisons		Coherence	Legibility	Refuge
Type 01	Type 02	***	***	NS
Type 01	Type 03	***	***	NS
Type 01	Type 04	***	***	NS
Type 01	Type 05	***	***	NS
Type 02	Type 03	NS	*	NS
Type 02	Type 04	NS	NS	NS
Type 02	Type 05	NS	*	NS
Type 03	Type 04	NS	NS	*
Type 03	Type 05	NS	NS	NS
Type 04	Type 05	NS	NS	NS

*Note*. Significance levels (*p*-value): *p < 0.05; ***p < 0.001; NS, not significant.

## Methodological limitations

Some particular indigenous plant species were not available in our database; thus, it is necessary to investigate these models with similar plant species. In terms of methodology, Wherrett (2000) indicated that it is critical to present multiple landscape components together to assess visual preference [27,28]. In order to obtain results by age, a more diverse sample of older and younger participants would be beneficial [8].

## Declaration of Competing Interest

The authors declare that they have no known competing financial interests or personal relationships that could have appeared to influence the work reported in this paper.
